# A Series of Cases, Exhibiting the Effects of Pressure in Cancerous and Other Diseases

**Published:** 1821-02

**Authors:** Samuel Young

**Affiliations:** Surgeon to the Cancer Institution, Gerard-street, Soho; Member of the Royal College of Surgeons, and of the London Medical and Chirurgical Society, &c.


### Original Communications, Select Observations, etc.

### *A Series of Cases, exhibiting the Effects of Pressure in Cancerous and other Diseases.* By SAMUEL YOUNG, ESQ. Surgeon to the Cancer Institution, Gerard-street, Soho; Member of the Royal College of Surgeons, and of the London Medical and Chirurgical Society, &c.

[Continued from page 31.]

THE next case to be reported (as noticed in the last published series of this Journal,) is taken from the minute-book of the Cancer Institution; and, although a case of very considerable standing, yet, as a great deal of aggravation had arisen from a comparatively recent injury, it was determined, in the first place, to try other probable remedies, for the relief at least of the increased state of pain, before the treatment by pressure should be adopted, so that the latter should be kept distinct, and its effects fairly appreciated unmixed with other efforts: and especially so, as there was evidently an impaired state of visceral functions with the patient, which very possibly augmented and kept up the painful state of the breast and arm; and which an alterative and active purgative plan would then most probably have relieved. In this case, however, it will be seen that an alterative plan, with active purging and occasional emetics, was pursued for three weeks without the slightest diminution of pain, and without the smallest amendment of any one symptom; but, on the contrary, that immediate relief was obtained upon the very first application of pressure, and the most permanent benefits followed the persisting in its use.

CASE.

*June* 10, 1819.—Lydia Elms, age 45; Grafton-street, Soho. Violent lancinating and gnawing pains of the whole left breast, but particularly at the nipple towards the inner side, and at the upper part; also about the shoulder and collar-bone. Great pain down the inner part of the arm, attended with swelling. No distinct tumor to be discovered, but an evidently diseased state of structure generally of the whole gland, particularly about the nipple, in the centre at the upper part of the breast, and the side leading to the axilla. Twenty-one years since, had a lump formed at the upper part of the breast, which came to matter and healed: then under the care of Mr. Birch, of Spring-gardens. After this, broke out several times, but ultimately healed. A slight degree of pain, with uneasiness, have ever since attended on the parts. Catamenia not subsided, though somewhat irregular. Health generally but very indifferent. Married, but never had any children.

*June* 11.—The great pain of the breast has existed for these three years; but, on the 25th of March last, on lifting a chest of drawers, the edge struck the breast, and all the symptoms since have been greatly aggravated; particularly the pain at the inner part of the breast by the nipple. Pil. alterat. decoct. tarax. mist. purg.

17.—The alterative pills have been taken every, and every-other, night, and the purging medicine very freely used. The decoct. tarax. was rejected by the stomach, though persevered in. The symptoms in no one instance relieved. The medicines to be actively continued.

22.—The medicines have been pursued, but no benefit has yet appeared. The breast and arm still very painful; nights very bad. Rep. med.

27. No relief. The medicines to be pursued, with the addition of emetics.

30.—No relief as to the pain of the breast or arm; but a better night on the 29th. After taking the emetic, a large quantity of bile thrown up. The medicines to be continued.

*July* 4.—No alteration. The stomach rejecting the dandelion decoction, as well as the food. The patient was directed to suspend the breast firmly with handkerchiefs. A light infusion of camomile and cloves, with the alterative pills, were ordered. The dandelion decoction to be discontinued.

7.—The stomach somewhat improved; and the breast a little easier, from the application of the handkerchiefs. Pressure to the breast and side by roller and compress. The last-ordered medicine to be continued.

13.—Pressure in general application to the breast and side has been uniformly and actively kept up. The patient states herself to be greatly relieved, indeed:—wonderfully so! to use her own expression. The pain down the arm particularly less; and, since the application of the pressure to the breast, the fingers of the left hand have much more freedom and use, which before were so stiff as to render the hand incapable of holding any thing. The patient can now use her fingers tolerably well, even to hold needle-work; before, she states her hand to have been perfectly useless.

Rest at nights. General health much improved. Pressure to be continued.

*August.*—The structure of the breast much reduced in its diseased thickness and morbid feel. Distinct schirrus round the nipple more perceptible. The pain in the arm subsided; free use of the fingers; and in every respect very much better. It was omitted by the patient to mention, when giving the history of her case, that, some three or four and twenty years ago, she had a distinct tumor in the same breast, (that is, previous to her going under the care of Mr. Birch,) and for which a removal by the knife was proposed by two surgeons in Buckinghamshire, where the patient then resided.

*October.*—The diseased state of the breast has greatly subsided. The patient has remained perfectly free from pain; has a free use of the hand and arm. An improved state of health; rest at nights; and a more confirmed and comfortable feeling generally.

This case has been submitted from time to time, with others, to the inspection of those medical gentlemen who favoured the author by their attendance on his Lectures, as already noticed.

*December.*—The patient's health retains its improved condition. The breast and arm have remained easy. The former diseased structure of the breast very obscure and difficult of detection.

*December* 1820.—The breast has remained, from under pressure or any kind of treatment, quite easy for many months. The use of the hand and arm strong and natural. The patient's health occasionally in a disturbed state.

Within the last two months, Dr. Pett, of Clapton, had a professional opportunity of examining the state of the patient's breast; and it may be well to remark, that I have since heen informed by a gentleman, in whose family the patient lived as an upper servant, that twelve years back, from the diseases state of her breast, she was then incapable of raising her arm to her head; and that the use of the whole limb was even then otherwise very much impaired.

Frequent notice has been taken of the treatment by pressure as a palliative, and it has been stated by myself, as well as others, that, even taken in the light of a palliative in advanced cases, as a palliative merely in a state of hopelessness and disease, that the treatment by pressure will not only be found superior to any other known method of practice; but, in so confined a view even, its application and right treatment will be found a valuable accession in the removal of pain, the prevention of further mischiefs, and the melioration generally of that misery which the advanced stages of the disease so frequently present.

This, however, has been a general statement from experience generally, without being illustrated by any individual cases. The following case from the minute-book is therefore given as an example, and may be taken as a fair average representation of this department of the practice:—*i. e.* the important effects of pressure in hopeless or irrecoverable cases, as a palliative remedy merely.

CASE.

*May* 21, 1819.—Ann Whitnell, age 55, a full corpulent habit; Wardour-street. A large cancerous mass or tumor occupying the whole space of the formerly-situated right breast, as well as the axilla; immovable on the parts beneath, and extending considerably round the side, and, for several inches below in front, taking the sweep of the ribs. The largely involved and discoloured integument loaded with tubercles. The morbid mass itself ragged and tucked in, and shooting out a considerable fungus.

The history and circumstances of the case were minuted down, from the patient's statement, as follows:

In March 1816, first discovered a drawing-in of the nipple, and, upon examination, found a large and very hard lump beneath. A twelvemonth previous to this, had had a discharge from the nipple of the same breast, but took no further notice of it. Not the least pain attended up to this period, and the patient was never more surprised in her life (as she says) to discover such an unlooked-for alteration. Upon the advice of Mr. Senior, she immediately went the next day to consult Mr. Astley Cooper; who prescribed for her, and ordered a plaster of soap cerate, and the application of three leeches once every week. The patient applied the leeches only once, on account of the irritation they produced, and the difficulty of healing the sores occasioned by their bites. In a few days after she consulted Mr. Travers, who prescribed for her; and shortly after she went to Mr. George Young, who also prescribed for her; but strongly objected to any excision of the tumor. During this time the disease was on the increase, and in the month of July a second tumor had formed on the side of the breast towards the axilla, and for the space of a month previously the whole had become very painful. The puckering of the breast considerably increased, and the nipple was quite lost. Although the parts had become much less movable, and the integument much more tightly stretched over the tumor, yet no discoloration of the skin existed.

The character of the pain was, sharp dartings and prickly shootings.

In the month of August following, (the disease and pain still on the increase,) she again consulted Mr. Travers; but any trial of the treatment by pressure, of which the patient had heard and proposed to submit to, being discountenanced from the first as useless, and (as represented) as producing insufferable pain, she submitted, and the parts were removed by the knife.

The wound after the operation healed in about three weeks; but the parts never returned to a natural state of feeling, and particularly under the arm much pain was experienced.

In about two months after the operation,—that is, in the October following,—a small white lump was observable in the cicatrix towards the side, which in the progress of eight weeks more became discoloured, and grew to the size of a large marble. (This tumor now is the size round of the large end of a hen's egg, and projects from the diseased mass about two inches or more, covered by a thin crimson cuticle.)

During this time the whole of the cicatrix became diseased and deeply puckered, particularly towards the side, the cavities of which were filled with small hard bodies. The growth of tumor, and advance of morbid alteration towards the clavicle, side, and axilla, now became evident, till it progressively arrived at its present formidable bulk and deeply and extended diseased state.

Within the last three months, the patient's health has become greatly impaired. Total loss of appetite, costive bowels, head-ach, with loss of flesh, and great weakness. Within this time also the right arm has become painful and enlarged, and at the present moment there is considerable swelling at the inner part of the arm above the elbow, and which the patient states is increasing. The patient was sent by me to Mr. Pearson for inspection. She was put for a few days on the alterative plan, and ordered to abstain from animal food.

*May* 25.—First application of pressure. Large commanding plaster-straps, compress, and roller. The compression produced, very considerable.

Up to the time of the application, the breast and side have continued very painful.

No inconvenience or additional pain experienced at the time the pressure was applied, and shortly after its application the former pain was considerably less, and the patient became (as she terms it) very comfortable. The pills had acted on the bowels. To be continued, with the dandelion decoction, daily.

*May* 26.—The breast and side quite easy; but the patient did not pass a good night, which she attributes to the novelty of the bandages. The breathing easy, which formerly had become impaired.

27.—Passed a very good night. Breast, &c. easy.

28.—The night not so good. Breast, side, and arm, quite easy; bowels costive. Magnes. sulph.

29.—A good night. Breast and side easy. The pain and swelling of the arm considerably better; the breathing much improved. The bowels have operated, though sparingly. The applications to the breast have remained, without any alteration, very firm.

This day an additional roller with compress was used, with great firmness. No pain. The patient thinks her general health is certainly better. Medicines continued.

*June* 1.—Removal of the compress and rollers, but the plaster-straps not touched. Re-application of linen compress and two rollers. No pain whatever produced by the application: on the contrary, the patient expresses herself as quite comfortable as to her breast and side, which have remained free from pain. The arm she also states as much improved, particularly as to the pain, shootings, and soreness from the elbow to the arm-pit, which have now quite subsided.

The size of the arm appears to be reduced to its natural state. The patient's general health is very bad; her stomach particularly deranged, with total loss of appetite. On Saturday experienced a violent pain across the part; but it has not returned, and altogether she thinks herself better to-day.

4.—The whole of the applications removed. Decided improvement has taken place. The entire bulk of the tumor much reduced. The formerly tightly-stretched integument can be taken up in folds. The disease so much reduced at the upper angle of the breast towards the axilla, that the patient can freely move her arm forward; which formerly, she states, was quite impeded by the bulk of the tumor at this part. Her arm also, she reports as quite natural; the pain of which used to deprive her almost entirely of rest at night; and which, through the day, in consequence of its great pain and uneasiness previous to the applications of compressure, she was constantly supporting in various positions for the last four months. In this state of suffering, the greatest relief the patient experienced was sitting between two low chests of drawers, and supporting her arms upwards on their tops. From all this now, however, she is entirely relieved.

The integument of the side, and its tuberculated diseased state in front below the breast, the patient also, from frequent previous examinations, considers much looser and otherwise improved; and which, surgically, is the fact. The large fungus seems to be much in the same state as before the application of the pressure, except that the covering cuticle is certainly less discoloured and otherwise diseased.

The only complaint the patient has made, is from the itching produced at the back, where the ends of the plaster-straps are placed. This has much annoyed her at nights. Although free from pain, and her rest and sleep considerably improved, the patient's general health is certainly inconsiderably changed for the better. She says that she faithfully follows the plan laid down for her; that is, abstaining in particular from any irregular sort of living.

Medicines as usual. Very active pressure employed, by the re-application of long plaster-straps, compresses, and two six-yard rollers. No pain.

*June* 25.—Progressive improvement of the disease; but the patient's general state of health on the decline.

N.B. The author's own indisposition, which confined him to his bed shortly after this period, gave an interruption to the further treatment of this case: but he has since understood that the patient had a severe bilious fever, from which she recovered, and had afterwards called at the Institution; that she had left her house in Wardour-street, and was supposed to have gone into the country.

The facts of this case, however, sufficiently speak for themselves, in establishing the treatment by pressure as a superior beneficial remedy, viewed as a palliative merely, without dwelling further on its merits, confirmed as they are in every case, upon a fair trial and right application of the practice.

There is one point, perhaps, proper to notice, connected with this case; and that is, the representation to the patient of the pain produced by pressure, previous to the removal of the disease by the knife, and which led to that operation. Of the integrity of that representation, however contrary to the known facts of the treatment in question, I am fully convinced; for at this moment I have a lady under my care, for cancer, with an aggravation of the disease, similar to the last-reported case, where great, indeed unlooked-for, benefits are now being derived under the treatment of compression, who some time since underwent the removal of the breast by the knife, and where Mr. Travers operated. The tight cording and rubbing, &c. of the bandages during the after-treatment of this case by

Mr. Travers, the patient represents as intolerable, and to which, in a great measure, she attributes the nervous irritability she has since laboured under; and which, upon the return of the complaint, greatly prejudiced her against any trial of the treatment by compression, thinking that the same, or rather an aggravated, degree of suffering would necessarily be produced, till the disease had made considerable ravages, before she submitted to the present treatment.

But this tight cording has nothing to do with the right application of compression to any given part; and it only leads to what has been over and over observed, again and again repeated, by others as well as myself, for the last five years, that this cording or bandaging of the trunk by Mr. Travers, or rather the usual bandaging of the London hospital-practice, is not the *new* method of bandaging by which the *new* method of treatment by *compression* is effected; than which nothing can be more opposite the one to the other, and producing in application the most opposite results.

Of the old method of bandaging the trunk in rib and collar-bone fractures and other diseases, I may have occasion to speak at some future opportunity, when endeavouring to afford a further illustration of bandaging the body upon right and efficient principles. It is only to be observed here, that a practice so barbarous, so devoid of all principle, as the old method of bandaging, can never be applied to carry in effect the compression of breast-diseases without producing mischief: a fact which the experience of the last five years has but too amply and practically established; and therefore those who endeavour to treat cases by pressure upon the old system of bandaging, may well fail, and represent the treatment as useless and as producing insufferable pain. But now there is sufficient information before the public, and enough has surely been said on the subject, for such palpable errors to be avoided.

CASE.

Richard Fiest, age 33, (of whose case the annexed plate affords a general appearance, as it existed in the month of December, 1815, when he first came under my care.) In a written statement of his case, this patient states that the disease at first was a small speck between the eye and the nose, the skin of which by accident was broken, by being struck by his nail in washing. This accident happened in 1803. "For this," the patient states, "I placed myself under a surgeon; but, getting no better, I went to another, but to no purpose: it kept spreading until it surrounded the whole eye, attaching itself to the bone in three different places, and growing very large and very painful, accompanied with almost constant bleeding,* and taking a course down the cheek until it contracted my mouth: all this time suffering without being informed what my complaint was, until told by Mr. Durbridge,† under whose care I then was, but without any success. From him, I was with Mr. M'Donald, Kent-road, all to no purpose, the disease still getting worse to worse. From him, to Mr. Astley Cooper for advice, who said, with an operation I might get well,—without it, never."

The patient could not, or would not, make up his mind to the operation, and returned again into the country; and in this way twelve years of his life were occupied, till he came under the present treatment.

A list of the different medical gentlemen, five in number, with their residence, for the sake of accuracy and proof, is given by the patient in his written statement; but it will be quite sufficient to state the fact generally, in order to show the nature and inveteracy of the disease, that the patient, previous to his coming under the present treatment, had contended with this malady for upwards of ten years, under the almost constant care of five or six medical gentlemen; till, ruined and worn down, he made a last effort to that source where a fame has been so long and widely established in forlorn and desperate cases, and he applied to Mr. Astley Cooper.

Although the disease had still continued to advance since the patient's application to Mr. Cooper, and a removal of the disease by the knife appeared to me wholly impracticable at the time of his coming under my care, yet, as an operation had been named, I thought it but fair to the practice in question that the practicability or impracticability of any removal by the knife should be decided by other competent judges, before the treatment of pressure was adopted. For this purpose I sent the patient to St. George's Hospital, where I met Mr. Brodie and Mr. Ewbank, who declared, after examining the case, that, from the extent of the involved integument, a thought of any removal by the knife could not then for a moment be enter-

* Fiest, in stating his case to me, has represented this bleeding as even copious at times. His trade was that of a boot and shoe maker, by which he (at Horsham) had brought up his father's family as well as his own, till totally ruined by the expense occurred on seeking relief for the disease of his face and eye. He says he could not even work as a journeyman, from the pain and bleeding: that, when he attempted it, with his head necessarily stooping, the pain became intolerable, and the blood would run down in a gutter on the floor. The integrity and honesty of his character is vouched for by the most respectable people in his neighbourhood, who have taken a great interest in his case; and it was through some friends of the late Lady Evelyn's that he came recommended to me.

† The patient was told his complaint was cancerous.

tained: at this interview Drs. Pearson and Young were also present.

Although the disease, particularly at the upper part, and of the upper-lid especially, (which was entirely converted into a darkish-brown crustaceous mass, or hard and rather brittle structure in parts, not unlike the diseased growth in Lea's lip, whose case was published in 1815, in the "First Minutes,") protruded so as to obscure all vision on that side; yet, when the under-lid was drawn down, or separated from under the projecting mass, and which was not so changed by disease as the upper-lid, the cornea of the eye then became visible, and the patient could discern objects. Upon any such attempts, the part was subject to bleed largely. In an undisturbed state, however, all appearance of eye or eye-brow, or any trace of the external appearance of the parts, was quite lost; the disease filling up and projecting beyond the cavity of the bony orbit, as generally represented in the accompanying plate.

The disease largely sprang from the inner angle of the eye; was firmly attached to the frontal bone just above the centre of the orbit, as well as at the outer angle, to the temporal bone.

The under diseased lid was almost obscured by the upper part of the disease; and the parts beneath covering the orbit were so morbidly changed and thickened as to obscure any feel of the bone beneath. The integument of the cheek towards the nose was tuberculated, and the corner of the mouth drawn upwards.

On the 16th of December, 1815, pressure was commenced by compress and roller, the bulk of the disease being a sufficient pad of itself, with a small quantity of lint only to fill up inequalities, and afford a general covering for the roller to produce the necessary compression. In the course of a few weeks, a considerable sloughing of the general mass of the disease had taken place; and in two or three months, the parts were evolved, and the eye-brow could now be seen, with detached portions of disease along its course, and the greatly-diseased mass which had once been the upper eye-lid hung beneath.

The disease of the under-lid, as well as that on the cheek, was also improved. A hollow at this time being formed by the sloughing of the disease from the orbit, the pad of a tourniquet covered with lint was now used as a compress.

Under this mode of treatment the disease still diminished. The discharge most offensive and acrid. It excoriated every part of the face with which it came in contact, and entirely destroyed the leather covering of the tourniquet pad in the course of a day or two. The sheet-lead also, which was afterwards used as a case to the pad and to enlarge the compress, was so acted upon by the discharge as to be reduced into small crumbling portions.

In the Easter following, his wife came to town to see the patient, and her first observation was, that "one side of his face was as long as the other." That is, the tuberculated disease of the cheek had been entirely removed by the pressure, and that side of the face, which had formerly been shortened, and the corner of the mouth drawn up in consequence, was now let down to its proper situation and length.

Some of these tubercles of the cheek sloughed out precisely the same as in the case of tuberculated integument in the cancerous female breast, when under similar treatment.

In the month of December, 1816, the disease of the upper eye-lid entirely came away; that is, the whole sweep of the former lid was removed, having only a few lines of diseased integument under the eye-brow, and which came in contact with the eye-ball. In this case, the eye, so far from projecting, was naturally sunk deep into the orbit. There was conjunctival disease at the inner angle.

The removal of the projecting disease, and ultimately of the disease of the upper eye-lid, afforded an opportunity of examining the state of the orbit itself; and the disease was found casing the cavity, as far as the examination of the finger and thumb could go. Under pressure, these diseased portions became detached from the bone; felt like cartilaginous plates of the thickness of a crown-piece, and ultimately disappeared under the pressure, the whole cavity of the orbit being filled up by graduated compress for the purpose. At the outer angle, disease still filled up the space between the eye and the orbit, so that the edge of the bone could not be felt. The fungous disease at the inner angle, the consistence of which was that of a gristly or cartilaginous flesh, had sloughed down, leaving a large cavity which took the first joint of the thumb to carry lint down to the bottom as a compress; the whole of the cavity, of course, being always so filled up.

The very thick fleshy feel of the under eye lid and integument filling up the under portion of the orbit, had greatly thinned away; but, on pulling down the under-lid, two or three crescent-like fleshy funguses were seen projecting upwards.

The integument of the cheek at the lower part had entirely recovered itself from the disease, and both in appearance and feel was quite natural.

After considerable removal of the disease had been effected, the patient was sent for the satisfaction of Mr. Astley Cooper's inspection; who stated, in the note with which he favoured the author in return, "that the disease was greatly improved, though a good deal of the old leven yet remained."

In the spring of 1817, the patient was unfortunately attacked by severe inflammation of the conjunctiva of the diseased eye,—a complaint very prevalent at the time. In the course of twenty-four hours, the conjunctiva, from being perfectly flat and white on the ball of the eye, rose considerably above the cornea, presenting a thick red ring, as if the eye had been set in red sealing-wax.

A complaint so sudden was considered entirely unconnected with the old morbid organization of the surrounding parts, and was treated accordingly. Pressure was suspended, and the complaint combatted in the usual way. The annular enlargement of the conjunctiva (which, totally distinct from the fungous production of the old disease, had a natural feel, and yielded to the edge of the lancet,) was freely scarified; lotions, with the opiate-wine, employed, as well as the wine itself in drops to the eye; and purgatives, emetics, with alteratives, were given.

The scarification of the conjunctiva did not seem to produce any good effect, but rather the contrary; and the patient always felt better, and the appearance of the eye was certainly improved, when the part was placed under general compression, the applications being kept damped by the lead and opiate wash. The attack gradually subsided; lotions of sulphat of copper were used, and subsequently the concentrated acetate of lead; but, though reduced, the annular enlargement of the conjunctiva did not entirely subside. The treatment by pressure was actively resumed against the remaining original disease.

The formidable portion of the disease at the inner angle had now given way; the deep cavity had filled up with healthy granulations; and skin was formed along the margin, as well as upwards from the sore partly along the nose, till the whole was quite healed, and presented an healthy sheet of skin close in to the very corner of the eye, the concavity of the part being perfectly natural.

In the autumn of 1818, the patient was considered as nearly recovered. The pressure had been discontinued frequently, to try the integrity of the parts either recently restored, or where disease had otherwise been removed. He had also occasionally returned to his home in the country during the treatment; and throughout had frequently been examined by Mr. Brodie and Mr. Ewbank, who had seen the disease before the commencement of the compression. A number of other professional gentlemen, from various parts of the country, had also seen the case during different periods of its treatment.

It may be proper here to remark, that, throughout the treatment, when general compression was employed to the part, neither uneasiness nor pain were produced, but rather the contrary: but, when the pressure was applied specifically to any point or angle, then the uneasiness and pain were frequently considerable; and a blowing sensation, like bellows; on these occasions was produced in the ears. The discharge, after the great sloughing of the disease *en masse* had subsided, became purulent, and lost its offensiveness. But, during the treatment, the same effluvia and discharge peculiar to the disease occurred at any time when a part, placed under specific pressure for the purpose, occasionally sloughed.

In the month of October of 1818, after the author had been absent for several weeks from town, he had the mortification to find that, from neglect and suspension of all pressure whatever, a considerable growth of disease had taken place. A firm fleshy substance projected from the centre of the eye at the under part, obscuring the ball; and at the outer angle a fig-like fungus, of an inch in diameter, and reaching down to the corner of the cheek-bone, with a broad and firmly-attached base, had projected itself.

Upon more minute examination, however, all the part at the inner angle, as well as the sweep of the eye-brow and other material points which had been formerly removed and healed, remained perfectly sound, and to all appearance quite healthy. This circumstance gave a hope that the disease at length might ultimately be conquered, or at least reduced to a very manageable state: and, with this hope, pressure was again actively employed to the new disease that had presented itself. This stage of the disease was frequently seen by Mr. Tegart, jun. of Pall Mall.

Under considerable interruptions of the treatment, the new disease was at length removed; but at the outer angle there was evidently disease filling up the space between the eye-ball and the bone. Pressure was actively and specifically thrown on this part; and the compress, from its shape, pressing on the edge of the bone, very considerable pain at times was occasioned.

During the author's Lectures in July and September in 1819, this case also came under the examination of Messrs. Haden and Leman, Dr. Armstrong, &c. The disease at the outer angle was not then perfectly cleared away; and the serrated edge of the integument under the eye-brow, and immediately in contact with the eye-ball, had rather a suspicious appearance. This part, trifling however in extent, and scarcely the thickness of a wafer, was constantly in a state of friction and moisture; and, from its situation, being immediately under the eye-brow within the orbit, and especially from its thinness, could not without difficulty be compressed. The annular enlargement of the conjunctiva remained as it had been during the last two years and a half, and formed for the eye a sort of lid at the upper part, against which the small portion of integument just described under the eye-brow was constantly rubbing, upon any motion of the eye, although to the patient it produced no sensible irritation or any other effect. For some months, pressure had only been employed at the outer angle, and that too under various interruptions. All the other parts remained healthy.

Under pressure, at length, the obstinate—or rather, from circumstance of situation, the hidden and defended—disease at the outer angle and interior of the orbit was perfectly cleared away; the bone was quite distinct, and the finger could be passed down between it and the ball of the eye.

Thus this man, with a numerous family, and exposed to extreme difficulties, pursued the treatment by compression, under a variety of interruptions and disheartening circumstances, through a space of four years, till he had conquered a disease which had engrossed seventeen years of his life. It may be right to observe, that, in a few months after the treatment was commenced, the patient was able to work at his trade, from which he had been wholly incapacitated by the disease for so many years previously.

In June, 1820, the patient, with his family, returned into the country; all pressure having been suspended between six and seven months.

In about three months afterwards, I was gratified to hear a satisfactory account of the patient, from a gentleman (Mr. Henry Tredcroft) resident in the neighbourhood, and who had taken great interest in the case from the beginning. In his letter of thanks to me on this occasion, he "regrets he could not get poor Fiest earlier under my care, as the cure would have been sooner effected." He also states, "I am sure it will add to your satisfaction to know that your kind attention has been bestowed on a person who well deserved it. His good character, added to his misfortune, will always secure from me an interest in his behalf. I shall at all times be ready and most happy to bear testimony to the ability and feeling displayed towards this poor sufferer, if ever I am called upon. I have seen more than one poor person in the last stage of this dreadful disease. I have seen their agony, and therefore feel particularly for those who labour under this complaint. Fiest is now living comfortably with his family; for which he is entirely indebted to you, &c."

It may be well to observe, in a practical point of view, that no bone, at least none that could be detected, exfoliated during the sloughing in Fiest's case: whereas, in Johnston's case, on the contrary, (the first reported in the present series,) a large portion of the bones of the eye and upper jaw was destroyed.

But it may also be proper to add, that Johnston's case was a disease that sprang from the bone, at least from the periosteum: whereas, in Fiest's case, the disease commenced in the soft parts, and only in progress became attached to the bones. In Johnston's case there was no original disease of the integument, the parts only becoming distended by the growth of the under tumor. In Fiest's case, on the contrary, the integument was wholly and originally converted and involved by the morbid growth, bearing every characteristic of the cancerous change; yet in this case there was no enlarged absorbent gland to be found in the neighbourhood of the disease, though five times the standing of Johnston's case, who had no one cancerous symptom of the integument, but where a cluster of enlarged glands did exist under the ear, at the angle of the jaw.

The deep-seatedness, as well as proximity, of the disease in Johnston's case, leaves little difficulty in the mind to conceive how the glands should become excited, if not ultimately involved in the same disease, although the patient had not been exposed, as he was, to severe cold and catarrhal affection: while in Fiest's case, on the contrary, it was a disease more of surface and projection, and distant from the seat of such glandular affection.

[*To be continued.*]

